# The Enigmatic Marine Reptile *Nanchangosaurus* from the Lower Triassic of Hubei, China and the Phylogenetic Affinities of Hupehsuchia

**DOI:** 10.1371/journal.pone.0102361

**Published:** 2014-07-11

**Authors:** Xiao-hong Chen, Ryosuke Motani, Long Cheng, Da-yong Jiang, Olivier Rieppel

**Affiliations:** 1 Wuhan Centre of China Geological Survey, Wuhan, Hubei, P. R. China; 2 Department of Earth and Planetary Sciences, University of California Davis, Davis, California, United States of America; 3 Laboratory of Orogenic Belt and Crustal Evolution, MOE, Department of Geology and Geological Museum, Peking University, Beijing, P.R. China; 4 Center of Integrative Research, The Field Museum, Chicago, Illinois, United States of America; University of Pennsylvania, United States of America

## Abstract

The study of the holotype and of a new specimen of *Nanchangosaurus suni* (Reptilia; Diapsida; Hupehsuchia) revealed a suite of hitherto unrecognized characters. For example, *Nanchangosaurus* has bipartite neural spines and its vertebral count is nearly identical to that of *Hupehsuchus*. It differs from the latter in having poorly developed forelimbs despite the advanced ossification in the rest of the skeleton. Other differences all pertain to hupehsuchian plesiomorphies retained in *Nanchangosaurus*, such as low neural spines. The relationship of Hupehsuchia within Diapsida was analyzed based on a data matrix containing 41 taxa coded for 213 characters, of which 18 were identified as aquatic adaptations from functional inferences. These aquatic adaptations may be vulnerable to the argumentation of character homology because expectation for homoplasy is high. There is an apparent incongruence between phylogenetic signals from aquatic adaptations and the rest of the data, with aquatic adaptations favoring all marine reptiles but *Helveticosaurus* to form a super-clade. However, this super-clade does not obtain when aquatic adaptations were deleted, whereas individual marine reptile clades are all derived without them. We examined all possible combinations of the 18 aquatic adaptations (n = 262143) and found that four lineages of marine reptiles are recognized almost regardless of which of these features were included in the analysis: Hupehsuchia-Ichthyopterygia clade, Sauropterygia-Saurosphargidae clade, Thalattosauria, and *Helveticosaurus*. The interrelationships among these four depended on the combination of aquatic adaptations to be included, i.e., assumed to be homologous *a priori* by bypassing character argumentation. Hupehsuchia always appeared as the sister taxon of Ichthyopterygia.

## Introduction

The Early Triassic saw the emergence of multiple marine tetrapod clades during the biotic recovery from the end-Permian extinction. Sauropterygia [1], which includes the Jurassic-Cretaceous plesiosaurs, and Ichthyopterygia [2], which eventually gave rise to dolphin-like ichthyosaurs, are the two major groups that appeared during this time period. Apart from these two major clades, smaller groups that may or may not be related to the two, such as *Omphalosaurus* [3] and Hupehsuchia [4] also emerged. Both of these groups are poorly known, and have been low in diversity.

Hupehsuchia is arguably the most bizarre marine reptile group of the Mesozoic, with a flat edentulous snout reminiscent of a duckbill and a heavily-built body with dorsal dermal ossicles. The group traditionally contained two monotypic genera, namely *Nanchangosaurus* Wang, 1959 [5] and *Hupehsuchus* Young and Dong, 1972 [6]. The two genera share many similarities, leaving some ambiguity about the distinction between them [4], [7]. A third form was recognized by [4] but has remained unnamed because the only specimen was incomplete, being mostly made of natural molds that are not well-defined in many places. A specimen similar to this form was reported by [8] but has again remained unnamed. Recently, a new monotypic genus was described under the name *Parahupehsuchus* [9]. This fourth form clearly differed from the first three, suggesting that the ecological and morphological diversity of the group was higher than previously thought.

Despite the potentially high diversity, Hupehsuchia is understudied as a whole, as is evident from the brief review given above that cited only six papers in total. Especially poorly known is *Nanchangosaurus*, which was restudied only once since its original announcement in 1959 by [4] in 1991,with less than two pages on its redescription. The lack of knowledge for *Nanchangosaurus* leaves the phylogenetic affinities of Hupehsuchia ambiguous because it is considered the most primitive hupehsuchian [9]. Several phylogenetic analyses of diapsid relationships including *Hupehsuchus* have been published [4], [10]-[14] but *Nanchangosaurus* has never been included despite its phylogenetic importance because of the lack of knowledge. Our recent observation of the holotype of *Nanchangosaurus* revealed several important features that were unrecognized before, thanks to the availability of improved lighting and microscope technologies. Most importantly, cranial sutures are better deciphered than before and some appendicular skeletal elements are recognized for the first time, allowing us to critically assess the shoulder and hip positions. The new knowledge enables us to analyze the phylogenetic affinity of Hupehsuchia based on *Nanchangosaurus* for the first time.

Wuhan Centre of China Geological Survey (WGSC hereafter) started a field excavation in Yuan'an County, Hubei Province, China in 2011 to investigate the evolution of marine reptiles in the Early Triassic. Yuan'an County is next to Nanzhang County, which includes the type locality of *Nanchangosaurus*. The two counties occupy different sides of the same mountains that yield these Early Triassic marine reptiles. Therefore, the geographic distance is minimal despite the difference in political division. The excavation resulted in about 10 new specimens of marine reptiles, one of which is reported here as the second specimen of *Nanchangosaurus*.

The new specimen exposes the lateral aspect of the skeleton for the first time, providing useful information about this enigmatic reptile. The purpose of the present contribution is to clarify the osteology of *Nanchangosaurus* based on the new specimen and the holotype, and reanalyze the phylogenetic position of Hupehsuchia among diapsids based on the new knowledge.

## Materials and Methods

### Specimens

The type specimen is accessioned at the Geological Museum of China, located in Beijing, China. Its specimen number is GMC V646, which has not changed since its initial description in 1959. The new specimen (WGSC 26006) was collected by WGSC in 2011 during a field excavation in Yuan'an County, Hubei Province, China. Proper permit was obtained from the Bureau of Land and Resources, China, for the excavation. The specimen is accessioned at WGSC, which holds a fossil collection and display at its main location in Wuhan City, Hubei Province, China.

### Phylogenetic data

Phylogenetic affinities of Hupehsuchia among Diapsida was analyzed using the software packages PAUP and TNT. We employed the morphological data matrix from [11] as the core data set of our analyses, with modifications and emendations as explained below. The matrix originally contained 188 characters coded for 34 taxa, of which four were non-diapsid outgroups. A similar data matrix is also available in [14]: the two matrices share a common root in [15] but have been modified by two different groups of researchers. We found the latter matrix to be less appropriate as the core data set for our purpose for the following reasons. First, there are fewer characters in this matrix (159 characters coded for 38 taxa), and many are sauropterygian-specific. Second, it contains fewer terrestrial diapsid taxa, while including many sauropterygians and similar forms, some of which are poorly-known. Third, it does not contain any outgroup taxa and character polarization is based on an hypothetical ancestor whose coding is zero in all features. This approach requires scrutiny of character polarization as new outgroup taxa are added to the fossil record. This potential hurdle may be circumvented by using explicit outgroups for character polarization.

The following modifications were made to the data matrix of [11]. We replaced Ichthyopterygia with its basal members, *Utatsusaurus* and *Chaohusaurus*, and added *Nanchangosaurus* and three additional marine reptile genera, namely *Largocephalosaurus* [14], [16], *Sinosaurosphargis* [13], and *Wumengosaurus* [12], [17], and two terrestrial genera, specifically *Pamelina* [18] and *Sophineata* [19]. We also added 25 discrete characters (Text S1), 11 of which were derived from [14]. We then replaced the presacral count (character 186) with the dorsal count. This is because the presacral count is partly redundant with the cervical count (character 187) that it contains, violating the independence of characters. We used the data compiled by [20] to code the dorsal count. We also replaced character 55, which codes interclavicle shape as rhomboidal or T-shaped, with a similar character coded for presence/absence of the anterior process of the interclavicle. This did not change the existing coding but allowed the addition of *Chaohusaurus*, whose interclavicle is neither rhomboidal nor T-shaped while retaining the anterior process. In total, there are 213 discrete characters coded for 41 taxa in the matrix (Text S1).

Character states were amended in the following parts of the data matrix. We employed the amendment by [18] of the character coding for lepidosaurs, which affected about 5% of the characters. Character coding for *Hupehsuchus* required an extensive revision based on the observation of specimens. It involved 62 characters, amounting to nearly a third of the original data set. Character states in other taxa were emended where appropriate, in nine of the 213 characters. See Text S1 for the list of emendations.

### Phylogenetic analysis

Heuristic searches in PAUP* 4.0b10 and TNT 1.1 were used for phylogenetic analyses. PAUP searches used 100 replicates of random additional sequences and TBR branch swapping, holding 10 trees at a time. New technology searches with the following options were used in TNT to confirm the result of PAUP: 100 replications, 100 drifts, and 10 multiplications. Bremer support was estimated using TNT. Bootstrap values are based on 10000 replicates and estimated again in TNT. Following the default setting, “?” was interpreted as missing data in both software packages.

To address potential biases from aquatic adaptations (see Discussion), we ran four sets of analyses. The first analysis was based on the raw data matrix without any treatment of aquatic adaptations. The second analysis tried to minimize the bias from aquatic adaptations by un-coding them as missing, i.e., “?” for marine reptiles. Taxon- and character-removal experiments constituted the third and fourth analyses.

The second analysis followed the steps below. First, we identified 15 of the 41 taxa to be marine reptiles that had limited locomotory ability outside of water, based on the possession of flipper- or paddle-shaped limbs—they are hupehsuchians, ichthyopterygians, thalattosaurs, saurosphargids, sauropterygians, and *Wumengosaurus*. Second, we identified 24 of the 213 characters to be aquatic adaptations based on functional inference. For example, buoyancy in water eliminates the need to support the body mass with limbs, so those limb features that are related to body support, such as the insertional crest for latissimus dorsi on the humerus (character 62), are expected to be reduced or lost in marine reptiles regardless of their phylogeny. Also, skeletal paedomorphosis, such as the reduction of pedal centralia [21], is commonly observed in marine reptiles, again probably because of the reduced gravitational constraint [22], [23]. See Text S2 for a complete list of the characters and reasoning. Third, we examined the character state distributions of these 24 characters to test if they are indeed seen across marine reptile clades with only limited exceptions. Six of the initial 24 characters were found not to be necessarily common across marine reptiles, leaving 18 characters as aquatic adaptations that would bias phylogenetic analyses. Finally, these characters were un-coded as “?” for those marine reptiles whose character states are considered to reflect aquatic adaptation because we lack the knowledge of the character states in the missing transitional forms. Un-coding of aquatic adaptation, rather than removal of characters containing aquatic adaptations as their character states, was employed because features resembling aquatic adaptation may evolve on land for reasons other than adaptation to aquatic lifestyles. For example, the thyroid fenestra in marine reptiles most likely reflects reduction of ossification from aquatic adaptation but a similar feature in lepidosaurs is clearly not an aquatic adaptation. The un-coding was applied only to marine reptiles for this reason.

In the third analysis, we removed one taxon at a time to investigate how their removal affected the most parsimonious topologies. Heterobathmy of characters, or ‘crossing of specialization’, exists naturally in phylogenetic data matrices [24] but excessive degrees of heterobathmy would result in polytomy in the strict consensus of most parsimonious solutions. The excess is often caused by a selected combination of taxa. The exercise of taxon removal aims to identify these taxa, if any.

The fourth analysis comprised character ‘removal’ experiments. We limited this exercise to the 18 aquatic adaptations, and instead of literally removing characters, we un-coded a selected set of aquatic adaptations as “?” at a time. We started from the character coding used in the second analysis, where all aquatic adaptations were coded “?”. We then selected a part of aquatic adaptations at a time and re-coded these characters back to the coding used in the first analysis. We tried all possible combinations of the 18 characters (n = 2^18^–1 = 262143; 1 was subtracted because the case where none of the 18 is included was already analyzed earlier), one at a time, and ran as many phylogenetic analyses using PAUP. The purpose of this exercise was to illuminate if any particular aquatic adaptation tended to cause all marine reptile clades to form a super-clade of most marine reptiles.

## Results

### Systematic Paleontology

#### Systematic hierarchy

Reptilia Laurenti, 1768 [25]

Diapsida Osborn, 1903 [26]

Hupehsuchia Carroll and Dong, 1991 [4]

Nanchangosauridae Wang, 1959 [5]

*Nanchangosaurus* Wang, 1959 [5]

*Nanchangosaurus suni* Wang, 1959 [5]

#### Holotype

**Figure 1:**
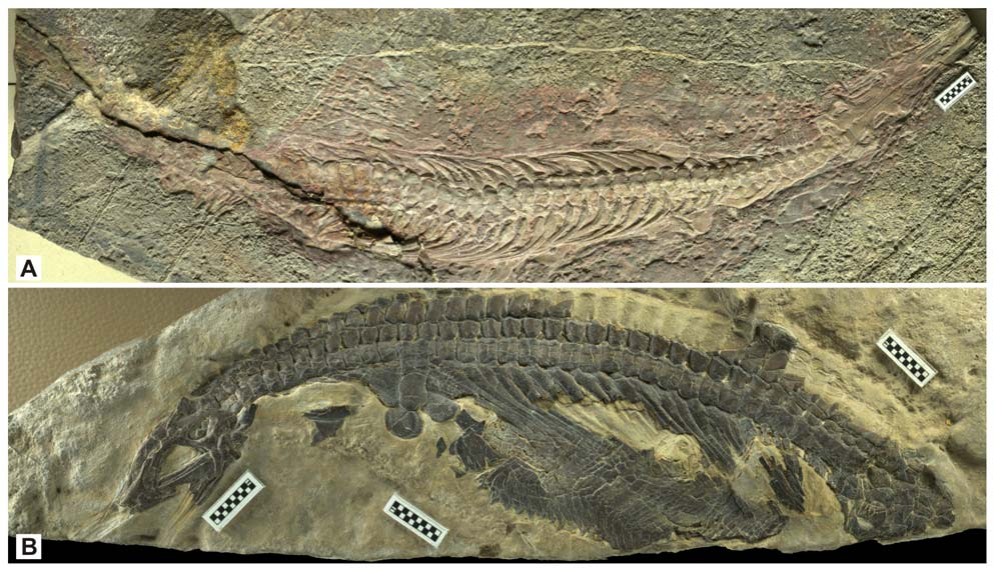
Skeletons of *Nanchangosaurus suni* Wang, 1959. A, holotype (GMC V636). B, referred specimen (WGSC 26006). Scale bars are 1 cm.

GMC V636 (Geological Museum of China, Beijing), a partial articulated skeleton exposed dorsally, lacking half of the snout, most of the appendicular skeleton, and part of the tail (Fig. 1A).

#### Referred specimen

WGSC 26006 (Wuhan Centre of China Geological Survey, Wuhan, Hubei, China), a partial skeleton exposed from left side, lacking most of the snout and tail (Fig. 1B).

#### Revised diagnosis

(Autapomorphies) forelimb poorly developed with short humerus, radius, and ulna; adult body size small, with presacral length of about 20 cm; (Plesiomorphies) single layer of dermal ossicles above dorsal neural spine; neural spines low.

#### Remarks

We did not find a unique feature in the skull of *Nanchangosaurus*, whose preserved part is very similar to that of *Hupehsuchus* except in size. However, *Nanchangosaurus* and *Hupehsuchus* are still considered as separate genera because *Hupehsuchus* shares synapomorphies with Parahupehsuchus that are lacking in *Nanchangosaurus* [9].

#### Locality and Horizon

*Nanchangosaurus* was initially reported to have occurred in the Daye Formation [5], which is now known to represent the Smithian and Induan (Lower Triassic). This led [4] to consider *Nanchangosaurus* stratigraphically older than *Hupehsuchus*, which occurs in the overlying Jialingjiang Formation of the Spathian (Lower Triassic). However, this information from the late 1950s has been outdated. It is known in the local community near the type locality of *Nanchangosaurus* that the type specimen of *N. suni* was discovered during the construction of a house, whose base rock belongs to the uppermost part of the Jialingjiang Formation. Therefore, *Nanchangosaurus* is coeval to *Hupehsuchus*, which occurs in the uppermost Spathian. The new specimen also occurred in the Jialingjiang Formation.

### Description

The two specimens are almost identical in size. The preserved length of the holotype is 28.4 cm, of which about 19.6 cm are precaudal. The referred specimen has a preserved length of 18.2 cm, all of which are precaudal. Both specimens seem to lack a similar proportion of the snout, so the precaudal length is estimated to be slightly above 20 cm. When assuming the body proportion of *Hupehsuchus*, the total length of *Nanchangosaurus* is marginally larger than 40 cm. This size is much smaller than in other hupehsuchians. *Hupehsuchus* has a precaudal length of about 50 cm or greater, whereas the same for *Parahupehsuchus* is much larger than 70 cm, although the missing skull prevents a reasonable estimation.

#### Cranium

**Figure 2:**
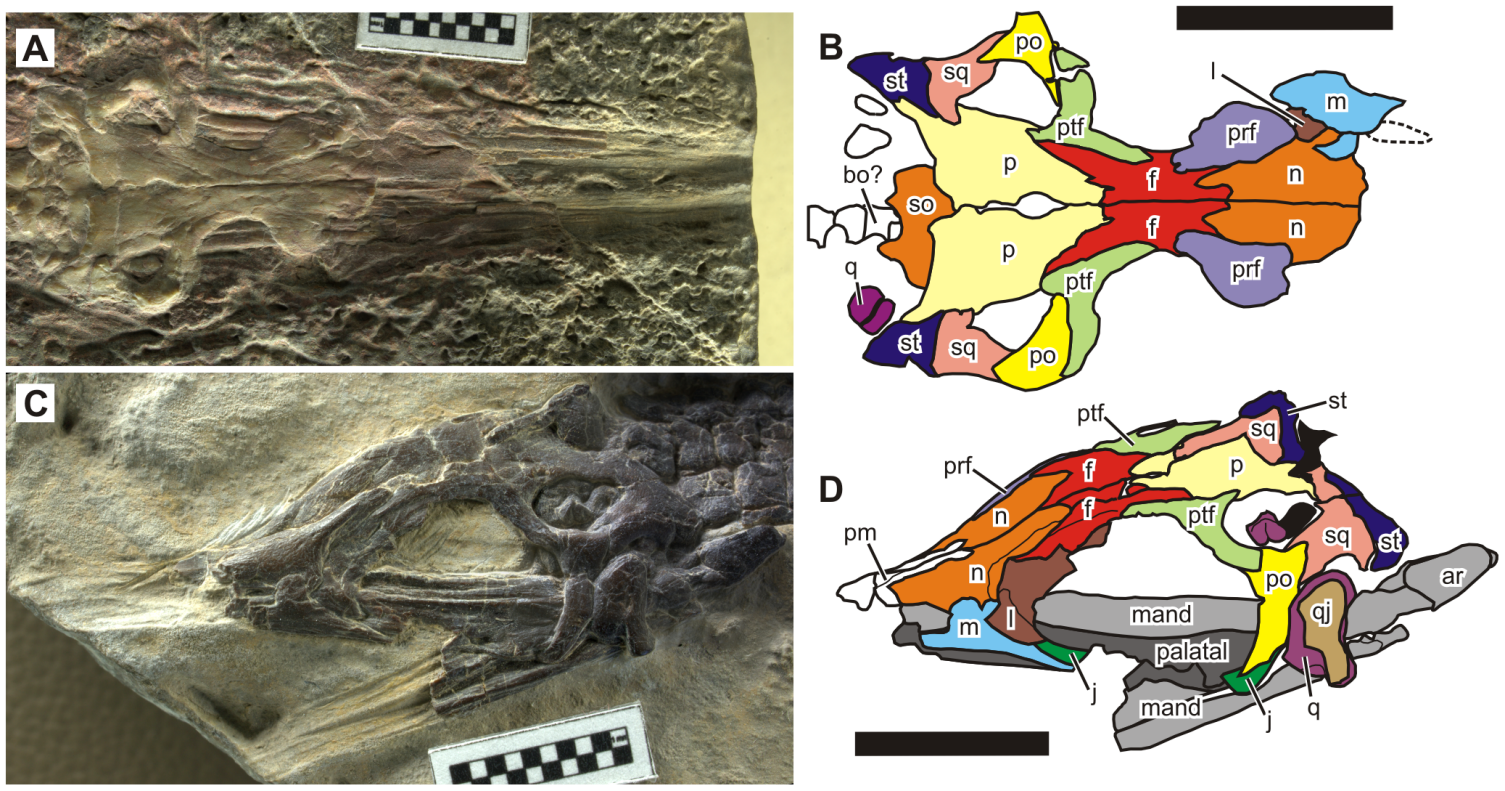
Skull of *Nanchangosaurus suni* Wang, 1959. A, holotype (GMC V636). B, referred specimen (WGSC 26006). Symbols: ar, articular; bo, basioccipital; f, frontal; j, jugal; l, lacrimal; m, maxilla; n, nasal; p, parietal; po, postorbital; prf, prefrontal; ptf, postfrontal; q, quadrate; qj, quadratojugal; so, supraoccipital; sq, squamosal, st, supratemproal. Colors: black, unidentified bones; dark gray, unidentified palatal bones; light gray, unidentified mandibular bones. Other colors are as labeled by symbols in the figure. Scale bars are 1 cm.

The skull is incompletely known because the snout is fragmentary in both specimens (Figs. 1, 2). A partial impression of the snout is present in the holotype (Figs. 1A, 2A) but it is not interpreted in Fig. 2B because of the difficulty in deciphering sutures with confidence. There is a pair of upper temporal fenestrae, each surrounded by the parietal, squamosal, postorbital, and postfrontal. The parietal bears a shallowly depressed shelf region laterally. The pineal foramen is large, and completely enclosed between the anterior halves of the paired parietals. The medial process of postorbital occupies a large proportion of the anterior margin of the upper temporal fenestra but a small participation of the postfrontal exists between the postorbital and the parietal.

The lower temporal fenestra lacks the ventral bar. In the referred specimen, the quadrate had been disarticulated from the squamosal and shifted rostrally (Fig. 2). In life, the bone was located more caudally, with an acute triangular embayment in front of it, representing the lower temporal fenestra that is open ventrally. The quadrate is largely overlapped by the quadratojugal, which is tall and narrow. The postorbital is more lunate than triradiate because its squamosal process is short.

The orbit is round, with its margin formed by the prefrontal, lacrimal, jugal, postorbital, postfrontal, and probably frontal (Fig. 2). The participation of the frontal in the orbital margin is clearly present in the holotype although very limited in its extent, as pointed out by [4]. In the referred specimen, however, the articular facet for the left prefrontal, clearly defined on the left frontal, suggests that the pre- and postfrontals probably met dorsally along the orbital margin in this laterally-exposed specimen. It is possible that the left postfrontal shifted slightly rostrally during compaction, making it appear as if it contacted the prefrontal. Similar shifting probably did not occur in the holotype because of its preservational posture that is dorso-ventral. Our preliminary observations suggest that the participation of the frontal in the orbital margin also depends on preservational postures in *Hupehsuchus*.

The supratemporal is located behind the squamosal (Fig. 2). In both specimens, it is excluded from the margin of the upper temporal fenestra by the squamosal, which instead occupies the entire posterior margin of the fenestra. This arrangement may appear unusual but is clearly present in both the right and left sides of the two specimens (i.e., four examples in total). The supratemporal is large, and bears a posterodorsal ‘lappet’ reminiscent of some ichthyopterygian supratemporals [27]. The socket for the quadrate is mostly formed by the squamosal, whereas participation of the supratemporal to this structure is obscure.

The external naris is incompletely preserved (Fig. 2). It is caudally bordered by a robust ascending process of the maxilla, which eliminates the lacrimal from the narial margin. The lacrimal exposure was narrower caudally than rostrally, as in basal neodiapsids: note that the missing prefrontal covered a large part of the lacrimal (Fig. 2D) in life. The nasal borders the dorsal side of the opening. The anterior margin is poorly defined but appears to be bordered by the premaxilla, which seems to have a robust supranarial process along the sagittal plane (Fig. 2D). This process lies medial to the nasal, and therefore does not seem to participate in the dorsal margin of the external naris.

The snout is elongated and broad. The snout impression in the holotype suggests that the preorbital part of the skull was longer than the rest. There is no indication of any dentition. It is most likely that this species was edentulous as is *Hupehsuchus nanchangensis*. The presence of a dental groove in the mandible of *Hupehsuchus* was suggested by [4]. It is difficult to confirm this feature in *Nanchangosaurus* given the state of preservation.

#### Axial Skeleton

There are 10 cervical and 26 dorsal vertebrae in the holotype, resulting in a total of 36 presacral vertebrae—see below for the justification of these numbers. The counts are 10, 27, and 37 in the referred specimen, respectively. These presacral counts exceed the number 34 previously suggested by [4]. Notably, our counts are very similar to the values known in the holotype of *Hupehsuchus nanchangensis*, which has nine cervicals and 28 dorsals, resulting in a presacral count of 37.

**Figure 3:**
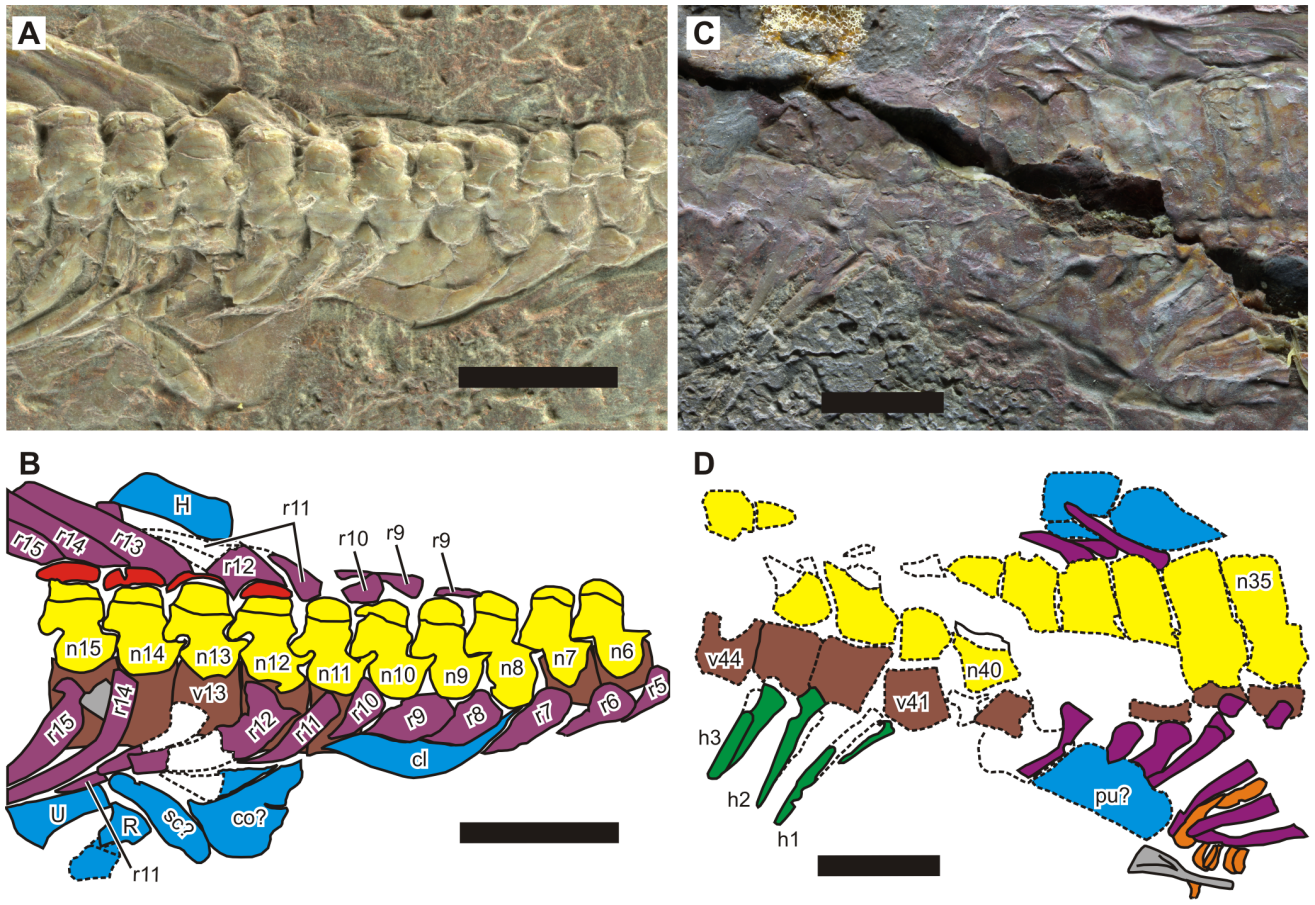
Pectoral and pelvic regions of the holotype of *Nanchangosaurus suni* Wang, 1959 (GMC V636). A–B, pectoral region. C–D, pelvic region. Symbols: Cl, clavicle; Co, coracoid; H, humerus; h, hemal spine and arch; n, neural spine; pu, pubis; R, radius; r, rib; U, ulna; v, vertebral centrum. Numbers associated with symbols represent vertebral counts with the atlas as 1. Colors: brown, vertebral centra; gray, unidentified bone; green, hemal arch and spine; light blue, appendicular skeleton; violet, rib; yellow, neural arch and spine. Scales are 1 cm.

We identified the most anterior dorsal vertebra as the one that bears the first elongated pair of ribs, following [4]. In the holotype, there is no clear evidence for rib elongation in the first 10 vertebrae, whereas the rib pair associated with the 11th vertebra is clearly elongated (Fig. 3A–B). The identity of the ribs attached to the 10th vertebra may be controversial because their tilt angles and narrowness are similar to those of the11th, unlike in the more anterior ribs that are broad. However, rib elongation cannot be positively identified in these ribs, either on the left or right side. We therefore consider them to be the last cervical ribs. The anterior end of the right clavicle is at the eighth vertebra, and the humerus is at the 13th (i.e., third dorsal vertebra). The first dermal armor element is seen above the 12th vertebra. The relevant counts are similar in the referred specimen—the most anterior rib pair with unequivocal elongation is at the 11th vertebra, the anterior end of the clavicle is at the ninth, the first osteoderm is above the 10th, and the humerus is at the 15th.

The sacral vertebrae were identified by a combination of two features: shortening and broadening of the ribs and the position of the pelvic elements. In the holotype, the shortest rib pair is associated with the 37th vertebra, and given its position relative to the suspected pubis and ilium (Fig. 3C–D), this pair is most likely sacral. The 38th pair is also broad, suggesting that they may be the second pair of sacral ribs. The ribs associated with the 36th vertebra may appear short on the right side because of its incomplete exposure; however, its left counterpart is longer and has a tapered end. Therefore, this pair is not sacral but the last dorsal. The position of the sacral vertebra is similar in *Hupehsuchus*. The position of the first hemal spine also supports the similarity of vertebral counts between *Hupehsuchus* and *Nanchangosaurus*—it is at the 41st and 42nd vertebrae in the holotype and referred specimen of *Nanchangosaurus*, respectively, whereas it is at the 41st in the holotype of *Hupehsuchus nanchangensis*.

**Figure 4:**
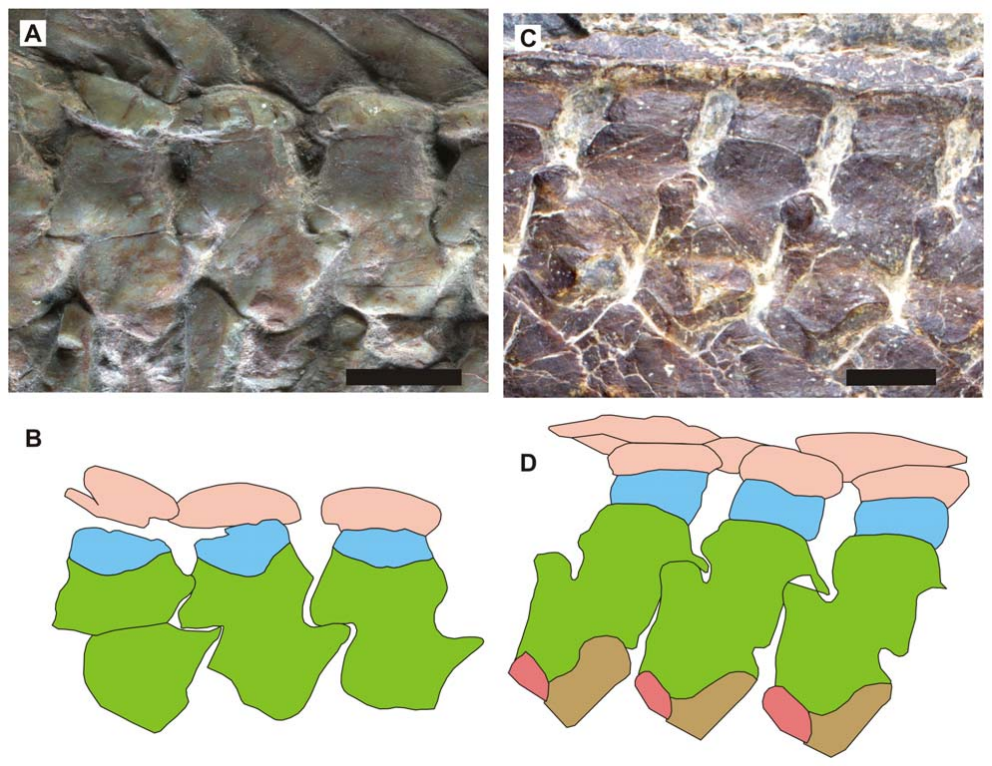
Neural spines and dermal ossicles of two hupehsuchians. A., *Nanchangosaurus suni*, based on the holotype (GMC V636). B, *Parahupehsuchus longus*, based on the holotype (WGSC 26005). Colors: brown, articular facet for rib; dark pink, articular facet for anterior rib; green, neural arch and first segment of neural spine; light blue, second segment of neural spine; light pink, dermal ossicles. Rib facets are not clearly exposed in GMC V636. Scale bars are 1 cm.

*Hupehsuchus* and *Parahupehsuchus* are distinguished by their strange neural spines that are bipartite (Fig. 4C–D), with a second segment atop the proximal neural spine. The dorsal segment is continuous with the first layer of dermal armor in *Parahupehsuchus* without a clear suture [9]. Such an extra segment was previously thought to be absent in *Nanchangosaurus* [4]. However, a close examination of the holotype under the microscope revealed that many of the dorsal neural spines had sutures between the proximal and dorsal segments. The sutures appear almost closed, and are very faint or absent in some of the dorsal neural spines. The referred specimen also has a short dorsal segment in at least some of the dorsal vertebrae. Apart from these two, the dorsal segments are mostly known from impressions left on the slab in the referred specimen, which is damaged dorsally (Fig. 1B). The neural spines are very low, unlike in *Hupehsuchus*. *Parahupehsuchus* also has low neural spines but the ones in *Nanchangosaurus* appear even lower (Fig. 4). Articulation of posterior dorsal neural spines is through small pre- and postzygapophyses, resembling the condition seen in terrestrial diapsids as described by [4]. This articulation was pointed out to be different from a strange articulation seen in *Hupehsuchus*, where the anterior margin of posterior dorsal neural spine is said to wrap around the posterior end of the neural spine that lies cranially [4]; however, we could not confirm such overlaps through preliminary observations of *Hupehsuchus* specimens.

**Figure 5:**
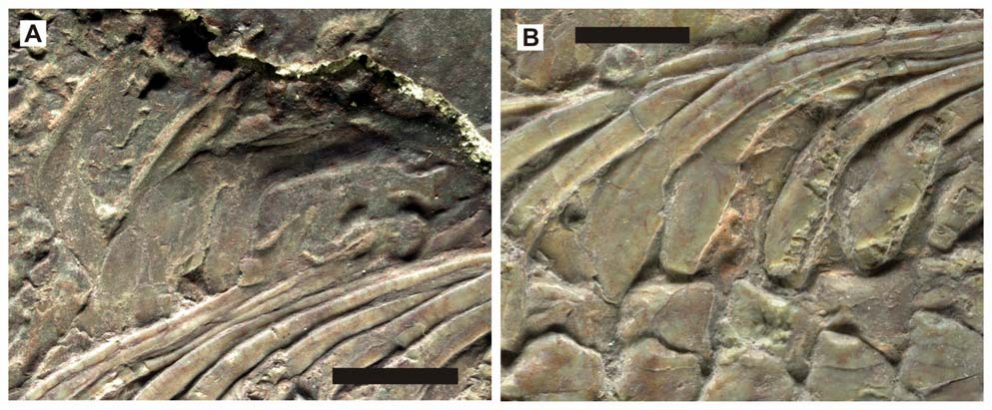
Two features of the axial skeleton of *Nanchangosaurus suni* Wang, 1959, discussed in text. A, lateral gastral elements of the holotype (GMC V636). B, posterior flange of dorsal ribs that exist proximally. Note that most posterior flanges are damaged, as evident in B. Scale bars are 5 mm.

Most of the dorsal ribs of the holotype are damaged proximally but some were spared the damage (Fig. 5B). The undamaged dorsal ribs reveal that the presence of a posterior flange proximally, sometimes overlapping the adjacent rib. The posterior flanges are better preserved in the referred specimen (Fig. 1B). Similar posterior flanges are known in *Hupehsuchus* and *Parahupehsuchus* [9] although they are distally more extended in the latter genus. The more distal part of the ribs of *Nanchangosaurus* are narrower than the proximal flange. This part of the rib seems to be flat and bears longitudinal striations, and may even have a longitudinal groove as in the holotype. In contrast, rib cross-sections of *Hupehsuchus* are rounder and almost elliptical in the mid-shaft region, which is smooth and has no longitudinal groove. The ribs are single-headed and articulate directly with the diapophysis at the end of a short transverse process of the neural arch. The rib heads are much narrower than the slightly more distal part of the rib with posterior flange, as in *Hupehsuchus* but unlike in *Parahupehsuchus* [9]. This suggests that the diapophysis alone is sufficiently wide to accommodate the rib head without help from parapophysis as in *Hupehsuchus* but unlike in *Parahupehsuchus*. The inferred absence of parapophysis cannot be confirmed because the lateral aspect is not exposed in any of the dorsal centra.

As noted by [4], lateral gastral elements are preserved in the holotype (Fig. 5A). They are flat and boomerang-shaped, as in *Hupehsuchus* and *Parahupehsuchus*, with the kink of the boomerang pointing cranially. The referred specimen preserves the lateral gastral elements in articulation, forming a complete wall (Fig. 1B). The elements overlap with each other for about a third of their widths, with the posterior element lying externally to the anterior counterpart. Several median elements are also exposed in the referred specimen. These elements have a shallow V-shape in overall appearance and round in cross-section, with the valley of V pointing caudally as in *Hupehsuchus* and *Parahupehsuchus*.

#### Appendicular Skeleton

**Figure 6:**
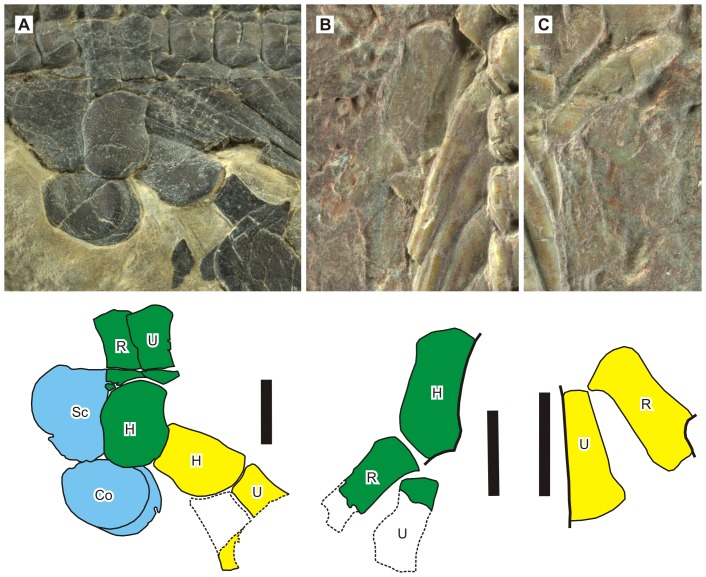
Appendicular skeletons of *Nanchangosaurus suni* Wang, 1959. A, referred specimen (WGSC 26006). B, left forelimb of the holotype (GMC V636). C, right forelimb of the holotype. Note the short and robust shapes of the limb elements. Symbols: Co, coracoid; H, humerus; R, radius; Sc, scapula; U, ulna. Scale bars are 5 mm.

Parts of the shoulder girdle and forelimbs are preserved in the holotype and referred specimen but only the three proximal bones of the forelimb are known (Fig. 6). The elements are incompletely exposed in the holotype (Fig. 6B–C). The humerus, radius, and ulna are all short and robust compared to those of *Hupehsuchus* and *Parahupehsuchus*, suggesting two possibilities: either these two individuals were immature or the limb skeletons were paedomorphic in this species. See below for further discussions of this issue.

Despite the shortness of limb bones, an anterior flange is easily recognized in the humerus, as in *Hupehsuchus*. The anterior margin of the flange is slightly concave despite the presence of the flange, again resembling the condition in *Hupehsuchus*. The surface striations suggest that the zeugopodial bones, especially the ulna, also have flanges off the shaft, where striations are radial and not parallel to the bone axis as would be expected in a long bone shaft (Fig. 6). There is a minimal space left between the radius and ulna in the referred specimen, whereas the bones are disarticulated in the holotype, preventing the confirmation of this feature.

The referred specimen exposes two coracoids and the left scapula. The coracoid is almost circular, and significantly smaller than the scapula. The scapula is incompletely exposed but it appears to be slightly expanded dorsally and crescent-shaped (Fig. 6A).

### Phylogenetic Analysis

**Figure 7:**
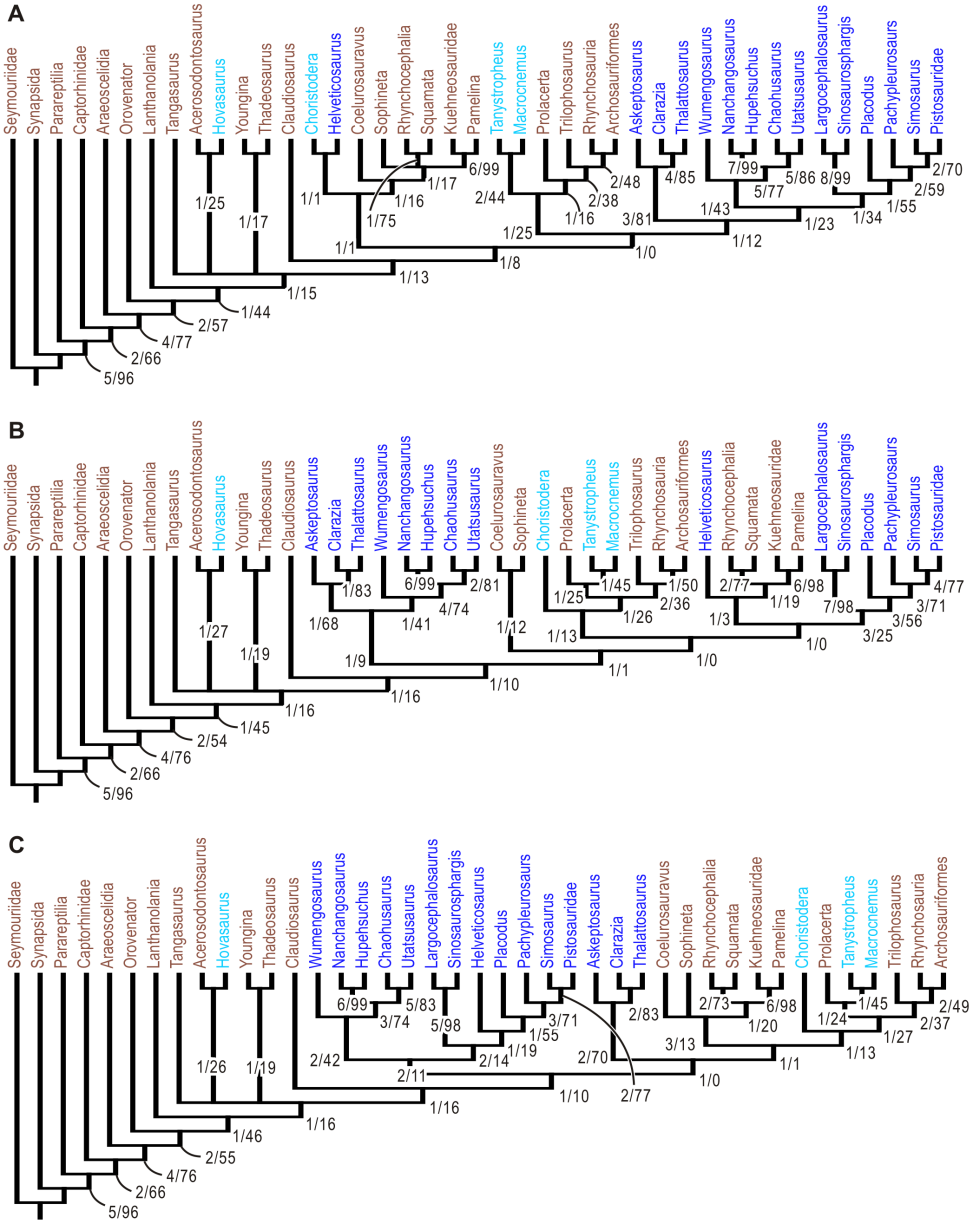
Strict consensus trees resulting from the phylogenetic analyses of marine reptile interrelationships. A, result of the first analysis, where all aquatic adaptations were considered ad hoc as homologies. B, preferred tree—result of the second analysis where aquatic adaptations in marine reptiles were un-coded as “?”. C, one of the results of the fourth analysis, where some aquatic adaptations were un-coded as “?”. Numbers associated with clades are Bremer support/Bootstrap value (n = 10000). Blue taxon names are for the 15 marine reptiles recognized (see text), light blue for semi-aquatic reptiles, and brown for the rest. All trees suffer from the lack of strong support in the middle of the tree.

The first analysis, which did not account for aquatic adaptation, resulted in four equally parsimonious trees (TL = 825; CI  = 0.315; RI = 0.610). The strict consensus of the trees combined 14 of the 15 marine reptiles identified earlier in one super-clade that lies next to the traditional archosauromorphs (Fig. 7A). The only exception was *Helveticosaurus*, which appeared on the lepidosauromorph side of Sauria. However, the examination of the nine unambiguous synapomorphies of the super-clade of marine reptiles revealed that seven of them were among the 18 aquatic adaptations identified earlier based on functional inference (Text S2)—they were characters 50, 62, 63, 114, 151, 180, and 186. The remaining two were characters 99 and 140. In contrast, each of the marine reptile clades within this super-clade were diagnosed mostly by non-aquatic synapomorphies.

The second analysis, where aquatic adaptations in marine reptiles were un-coded as “?” in marine reptiles, resulted in 2 equally parsimonious trees (TL  = 792; CI  = 0.321; RI  = 0.586)—note that the CI value is biased because some characters containing aquatic adaptations became phylogeny uninformative after un-coding of relevant marine reptiles. The strict consensus of these trees is given in Fig. 7B. The super-clade of all marine reptile is not recognized but the monophyly of each marine reptile clade was supported. Three different marine lineages are recognized, namely, *Helveticosaurus*, Sauropterygia and Saurosphargidae, and a large clade containing Ichthyopterygia, Hupehsuchia, *Wumengosaurus*, and Thalattosauria. The first two appeared on the lepidosauromorph side of Sauria, whereas the last one was located outside of Sauria. Within this last clade, Ichthyopterygia and Hupehsuchia formed a clade, with *Wumengosaurus* as the sister taxon.

**Figure 8:**
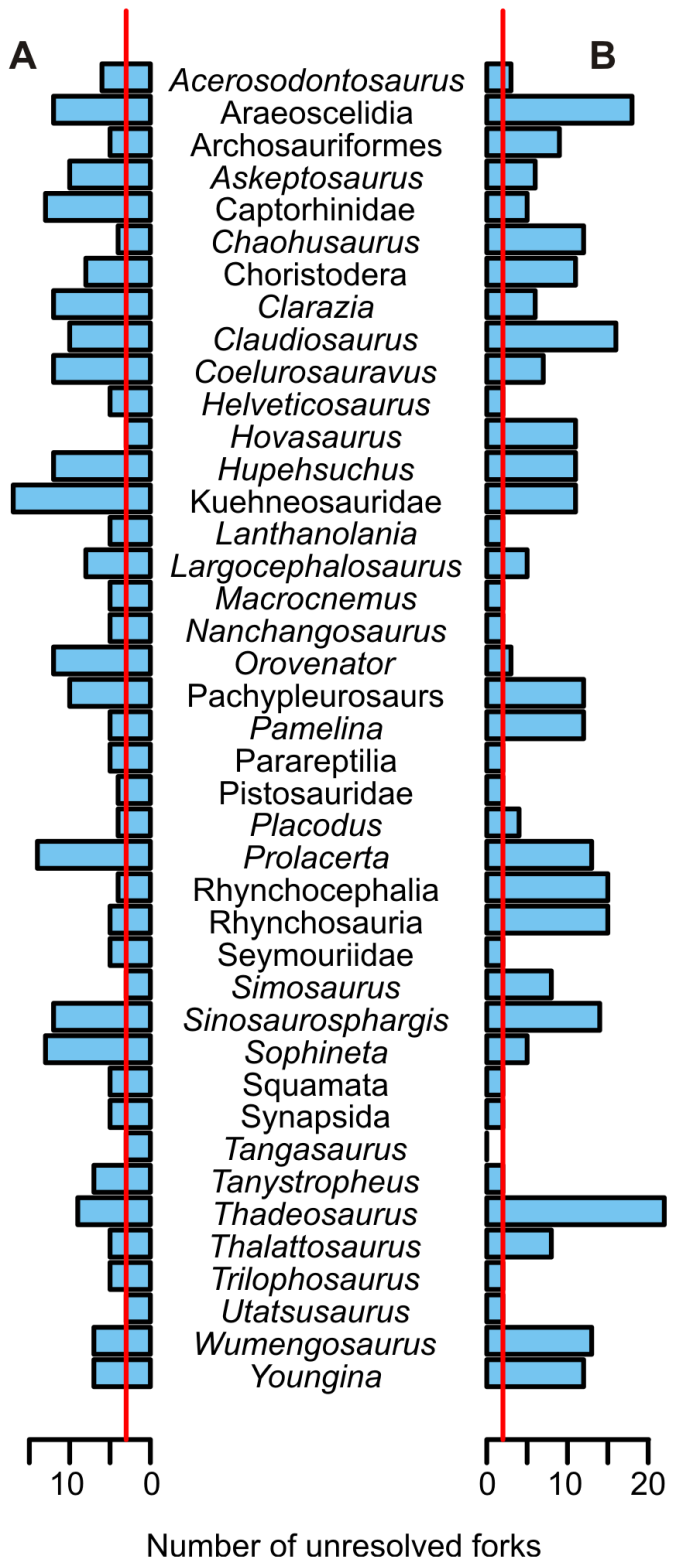
How removing a single taxon from the data matrix alters the resolution of the resulting strict consensus tree. A, removing a taxon from the raw data matrix used in the first analysis. B, removing a taxon from the data matrix of the second analysis, where aquatic adaptations are un-coded as “?”. Red lines indicate the number of unresolved forks (polytomies) resulted from the original matrices before taxon removal. Taxon removal usually resulted in increased polytomies than did the original matrices.

The third analysis, which removed one taxon at a time to examine the effect of each taxon, resulted in various degrees of polytomies in the strict consensus tree, depending on the taxon that was removed. The results are summarized in Fig. 8. Removing a taxon usually resulted in polytomies. Extreme polytomies were observed when removing a certain taxon, such as Araeoscelidia, Kuehneosauridae, *Prolacerta*, or *Thadeosaurus*, resulted in extreme polytomies among neodiapsids. This suggests that there is a high degree of incongruence in the data, and almost every taxon is needed for the trees in Fig. 7 to obtain. There were a few exceptional taxa: removing *Utatsusaurus* did not affect the resolution of the strict consensus tree, and deleting *Tangasaurus* reduced the number of polytomies.

**Figure 9:**
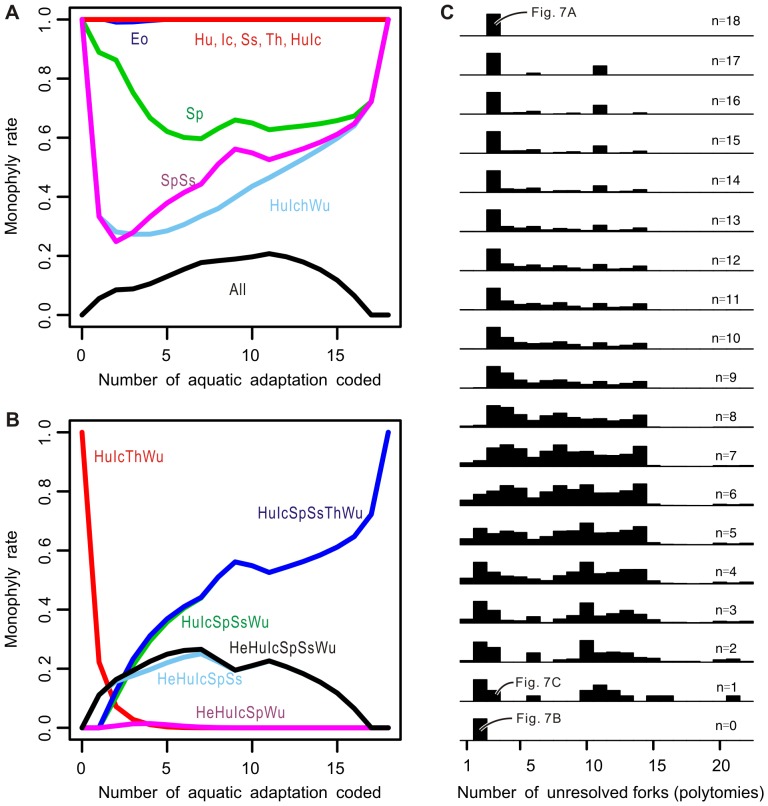
Effects of increased number of aquatic adaptations included in phylogenetic analysis. A and B, frequency at which a given clade appears monophyletic in the strict consensus tree of the most parsimonious topologies. The x axis represents the number of aquatic adaptations included (coded) in the analysis, i.e., x = 18 is the raw data whereas x = 0 is when all aquatic adaptations are un-coded. C, histograms of the frequency of the number of unresolved forks (polytomies) found in strict consensus trees (y-axis), and how the distribution changes with the number of aquatic adaptations coded in the data matrices (x-axis). Curves in A and B are identified by combination of taxon names, which are abbreviated as: All, all marine reptiles; He, *Helveticosaurus*; Hu, Hupehsuchia; Ic, Ichthyopterygia; Sp, Sauropterygia; Ss, Saurosphargidae; Th, Thalattosauria; Wu, *Wumengosaurus*. For example, SpSs indicates a clade comprising Sauropterygia and Saurosphargidae, whereas Sp, Ss represents each of Sauropterygia and Saurosphargidae, respectively.

The fourth analysis led to the following eight observations in strict-consensus topologies. First, the monophylies of individual marine reptile clades, namely Eosauropterygia, Hupehsuchia, Ichthyopterygia, Saurosphargidae, and Thalattosauria, were each supported almost regardless of the treatment of aquatic adaptations (Fig. 9A, blue and red lines). The only exception is Sauropterygia, which sometimes disintegrated into Eosauropterygia and Placodontia (Fig. 9A, green line). Second, Ichthyopterygia and Hupehsuchia always formed a clade regardless of the treatment of aquatic adaptations (Fig. 9A, red line). Third, this clade and *Wumengosaurus* formed a clade in Fig. 7A and B but the clade may be absent when an intermediate number of aquatic adaptations are coded (Fig. 9A, light blue line). The clade comprising Saurosphargidae and Sauropterygia also had a similar tendency (Fig. 9A, magenta line). Fourth, monophyly of all marine reptiles only occurred when certain combinations of aquatic adaptations were added, although its occurrence was generally rare (Fig. 9A, black line). When it occurred, the interrelationships among marine reptile clades were usually unresolved within this large clade, leading to a basal polytomy. Fifth, the super-clade that contained all marine reptiles but *Helveticosaurus* resulted with higher frequency as more aquatic adaptations were added (Fig. 9B, black line). For the super-clade to appear, it was necessary to have one of the following five aquatic adaptations coded in the matrix, together with at least another aquatic adaptation: characters 1, 58, 63, 68, or 180. This condition, however, was not sufficient because inclusion of such aquatic adaptations did not always result in the super-clade. For example, the super-clade was not formed even when 17 of the 18 aquatic adaptations were coded, as long as one of the following 5 characters was un-coded: characters 58, 63, 68, 71, or 180. Sixth, certain combinations of aquatic adaptations, when added, led to a well-resolved tree topology (Fig. 7C) that was different from the two end-member topologies (Fig. 7A, B). The Sauropterygia-Saurosphargidae clade, rather than Thalattosauria, became the sister group of the Hupehsuchia-Ichthyopterygia-*Wumengosaurus* clade in this topology (Fig. 9B, green line), without Thalattosauria joining the clade. Seventh, Thalattosauria and *Helveticosaurus* were rarely found in the same marine clade. Eighth, the inclusion of aquatic adaptations initially increased the frequency of polytomy in the strict consensus of most parsimonious solutions but then the frequency decreased again, eventually returning to the original level as even more aquatic adaptations were added (Fig. 9C).

## Discussion

### Anatomy of *Nanchangosaurus*

It has been thought that *Nanchangosaurus* had a shorter body than *Hupehsuchus* [4]. The same paper also suggested that the following features were found in *Hupehsuchus* but not in *Nanchangosaurus*: the unusual posterior-dorsal zygapophyseal articulation; second element in dorsal neural spines; and lack of frontal participation in the orbital margin [4]. However, our examination revealed that there was little difference between the two genera in these features, as described above. Despite such similarities, morphological differences do exist between *Nanchangosaurus* and *Hupehsuchus*, as pointed out by [4]. Unlike *Hupehsuchus*, *Nanchangosaurus* has poorly developed forelimbs, low neural spines, and only one layer of dermal ossicles above dorsal neural spines instead of three. Most of these features are plesiomorphic to Hupehsuchia [9], leaving the poor development of limbs as the only autapomorphy recognized for the monotypic genus.

The underdeveloped forelimb skeletons in *Nanchangosaurus* suggest a possibility that the two individuals described here may be immature and not paedomorphic. This possibility was discussed by [4], who concluded that such was unlikely. We agree with their suggestion that it was unlikely for the neural spine of *Nanchangosaurus* to grow much taller. The new evidence from *Parahupehsuchus* suggests that a low neural spine is a plesiomorphic feature of Hupehsuchia [9], so the very tall neural spines found in *Hupehsuchus* is an apomorphy. Moreover, the fusion between the first and second segments of neural spine is more progressed in *Nanchangosaurus* than in *Hupehsuchus*, whereas the opposite would be expected if *Nanchangosaurus* specimens were immature. The degree of development of the posterior flange of dorsal rib is nearly identical between *Hupehsuchus* and *Nanchangosaurus*, again suggesting that *Nanchangosaurus* specimens are mature. It would be ideal to find immature specimens of both genera to fully establish the relative maturity of the specimens described here.

When ribs and gastralia of *Nanchangosaurus* are combined, they constitute a robust rib basket that is heavily ossified except some dorsal intercostal space. The construction is similar to the body tube of *Parahupehsuchus* [9] but the double rib articulation that solidifies the trunk of the latter is absent from *Nanchangosaurus*. This lack and the intercostal space in *Nanchangosaurus* probably allowed more trunk flexibility than in *Parahupehsuchus*. This is witnessed by the preservational posture of the holotype, whose trunk is essentially straight but slightly curved in dorsal view (Fig. 1A).

### Phylogenetic implications

The premise of phylogenetic systematics is that character congruence under total evidence can test alternative tree topologies [24]. However, character statements that form the basis of such a test are hypotheses themselves, although they often remain untested [28]. The lack of such tests strongly impairs the causal groundings of cladistic analysis, so it is necessary to evaluate inherent, developmental, and functional causes behind character statements in an attempt to test them [28]. In the present case, such a test is especially important. The single most parsimonious tree resulting from the original matrix of [11] was weakly supported by the data near the middle part of the tree where basal neodiapsids were located, as is evident from a very low Bremer support value of only 1 found across the nodes in the area. Collapsing these weakly supported nodes, which is similar to finding the strict consensus of trees that are one step longer than the most parsimonious solutions, would result in a largely bush-like topology among basal neodiapsids. This suggests that there is a high degree of incongruence among characters, and the tree topology is vulnerable to poorly-tested character statements and the assumption of homology therein.

Given this vulnerability, the outcome of the phylogenetic analyses presented above needs to be interpreted carefully. The difficulty lies in the interpretation of aquatic adaptations, which may have evolved convergently in different clades for a common cause of adaptation to aquatic lifestyles [4], [29], although they may be homologous among multiple marine reptile clades. These characters have undeniable expectations for being homoplastic between a given pair of marine reptile clades than other characters. Then, it may not be justified to assume that they are all homologous *a priori*. Moreover, the fossil record of early transitional forms for most marine reptile clades is largely incomplete, so aquatic adaptations in derived members preserved in fossils may appear as homologies across marine reptile clades even when they are in fact homoplastic—a recent discovery of the centralia in a basal ichthyopterygian serves as an example of such risk [21]. A similar case of functional bias in phylogenetic reconstruction is known in limb-reduced squamates adapted for burrowing [30]. Also, it has been shown in salamanders that inclusion of paedomorphic characters in phylogenetic analyses, in association with aquatic adaptations, resulted in incorrect trees that were statistically well-supported in both parsimony and Bayesian analyses [31]. These cases encourage the character removal experiments of aquatic adaptations undertaken in this study.

Fig. 7A and B are based on two extreme assumptions of the nature of aquatic adaptation: Fig. 7A assumes that all are homologous bypassing the test of character statement, whereas Fig. 7B presumes that all are more likely homoplastic than homologous as a result of such a test, strictly enforced. The reality is expected to lie somewhere in-between the two. One of such intermediate results is Fig. 7C. These phylogenetic hypotheses are far from conclusive because Bremer support values for many of the nodes are only 1 step. Additional neodiapsid fossils from the Upper Permian and the Lower Triassic, especially of terrestrial and amphibious forms, may help resolve the problem.

Despite the generally weak Bremer supports and the discrepancy among the trees given in Fig. 7, certain robust inferences can be made from the results of the phylogenetic analyses. First, the monophyly of each marine reptile clade, namely Hupehsuchia, Ichthyopterygia, Sauropterygia, Saurosphargidae, and Thalattosauria, is well-supported, regardless of the treatment of aquatic adaptations. We therefore consider them valid. Second, the clade comprising Hupehsuchia and Ichthyopterygia is also valid, given that high support for this clade is present in all three trees in Fig. 7, and that the clade consistently resulted throughout the 262,143 iterations of the fourth analysis (Fig. 9A, red line). Third, two more clades that are present in all three trees of Fig. 7C are probably valid, namely the clade comprising Hupehsuchia, Ichthyopterygia, and *Wumengosaurus*, as well as another clade consisting of Saurosphargidae and Sauropterygia. These clades did not appear in the strict consensus trees when intermediate numbers of aquatic adaptations were coded in the data matrix (Fig. 9A, light blue and magenta lines). Even in such cases, however, 50% majority consensus trees contained these clades. These clades, therefore, are better supported than other large clades that comprise multiple marine reptile clades. When accepting these results, Triassic marine reptiles are most likely divided into four clades, namely *Helveticosaurus*, Hupehsuchia-Ichthyopterygia-*Wumengosaurus*, Sauropterygia-Saurosphargidae, and Thalattosauria. The grouping of Hupehsuchia and Ichthyopterygia is also supported by an observation that at least two Jurassic ichthyosaur specimens have bipartite neural spines, which may be an accidental expression of genes inherited from a common ancestor of Hupehsuchia and Ichthyopterygia [32].

The validity of other inclusive clades of marine reptiles is less certain. The super-clade of all marine reptiles but *Helveticosaurus* only appears under certain conditions, as detailed in the section for Results and seen in Fig. 9B (blue line). The clade comprising Hupehsuchia, Ichthyopterygia, Thalattosauria, and *Wumengosaurus* is present in Fig. 7B but this clade quickly disappears as a small number of aquatic adaptations are coded in the data matrix (Fig. 9B, red line) and never re-appear. Similarly, the clade of all marine reptiles except Thalattosauria appears in Fig. 7C but it is not formed when all or no aquatic adaptations are included (Fig. 9B, black line). Four of the 18 aquatic adaptations appear to be particularly important in forming the super-clade of all marine reptiles but *Helveticosaurus*. They are: coracoid foramen between coracoid and scapula (character 58, state 1), humerus epicondyle reduced (character 63, state 1), thyroid fenestra present (character 68, state 1), and nares located in the middle of the snout or more caudally (character 180, sate 1)—see Text S2 for more precise definitions and the reasons why they are considered aquatic adaptations. Two additional aquatic adaptations seem to play some roles in the formation of this clade: premaxilla enlarged (character 1, state 1) and intertrochantric fossa rudimentary or absent (character 71, state 2). Note that characters 1 and 180 both concern snout elongation and have almost identical character state distributions, with the only difference found in semi-aquatic protorosaurs, i.e., snout elongation of marine reptiles is being counted twice in effect by including both characters.

The phylogenetic position of marine reptile clades within Diapsida also varied depending on the treatment of aquatic adaptations. When the super-clade of all marine reptiles but *Helveticosaurus* is formed based on features interpreted as aquatic adaptations (Fig. 7A), it is placed basally to archosauromorphs, with *Helveticosaurus* joining lepidosauromorphs. When all aquatic adaptations are un-coded (Fig. 7B), the Sauropterygia-Saurosphargidae clade appears basally among lepidosauromorphs, outside of *Helveticosaurus*. The Hupehsuchia-Ichthyopterygia-Thalattosauria-*Wumengosaurus* clade lies outside of Sauria in this case. In Fig. 7C, all marine reptiles are outside of Sauria, even including *Helveticosaurus*. Fig. 7B accords well with existing hypotheses that Sauropterygia belongs to lepidosauromorphs [1], and that Ichthyopterygia lies outside of Sauria [33]. In most cases, marine reptile clades of various combinations are attached to one or more of the three internodes that connect to the last common ancestor of Sauria.

Aquatic adaptations, which led to Fig. 7A, have different phylogenetic signals than the rest of the features, which supported the topology in Fig. 7B, and tend to bundle multiple aquatic clades together. The effect of increasing numbers of aquatic adaptations in the analysis is evident in Fig. 9C. The incongruence among the characters rises as more aquatic adaptations are added to the data, inflating the number of unresolved tree forks (i.e., polytomies) in the resulting strict consensus trees (Fig. 9C). Once sufficient numbers of aquatic adaptations are added, however, they override the existing phylogenetic structure and join all marine reptile clades except *Helveticosaurus* in one super-clade (Fig. 9A and C), without much help from non-aquatic features. As this super-clade starts to appear more frequently, the number of polytomies decreases again. There is at least some bias arising from the excessive number of aquatic adaptations in the data relative to other features characterizing the relevant part of the tree.

Similar biases are expected if a marine reptile clade is used as an outgroup of another marine reptile clade when analyzing the internal topology of the latter (e.g., using Ichthyopterygians as the outgroup for an analysis of sauropterygian phylogeny). The only exception would be Hupehsuchia and Ichthyopterygia, whose sister group relationship appears well-supported by evidence, as shown above. In addition, it is probably justified to include Saurosphargidae as the sister taxon of Sauropterygia, and *Wumengosaurus* as the outgroup of Hupehsuchia-Ichthyopterygia. Inclusion of *Wumengosaurus* in the analysis of sauropterygian phylogeny may mislead the outcome.

## Conclusions

*Nanchangosaurus* is characterized by a uniquely underdeveloped forelimb and other characters that are likely shared with the outgroup. It resembles *Hupehsuchus* in more features than previously thought, including the bipartite neural spines, vertebral counts, and the posterior flange of dorsal ribs. It is smaller than *Hupehsuchus* but unlikely to be a juvenile specimen of the latter.

Phylogenetic analysis of hupehsuchian affinities among diapsids is complicated by inferred aquatic adaptations, which tend to bundle multiple marine reptile clades together against the phylogenetic signals from all other characters. The outcome depends on how many and which aquatic adaptations are assumed to be homologous *a priori* by bypassing character argumentation. There is a tendency for a large inclusive clade of marine reptiles appearing as more aquatic adaptations are included in the analysis.

Individual marine reptile clades, namely Hupehsuchia, Ichthyopterygia, Sauropterygia, Saurosphargidae, and Thalattosauria, are derived without any aquatic adaptations, whereas larger groups containing multiple marine reptile clades only appear when certain combinations of aquatic adaptations are included in the analysis, with two exceptions. Hupehsuchia is a sister clade of Ichthyopterygia, and *Wumengosaurus* is probably their sister taxon, regardless of aquatic adaptations. Also, Sauropterygia and Saurosphargidae likely form a clade. No clear conclusion can be given at this point on whether even larger clades of marine reptiles should be considered valid. The phylogenetic position of marine reptile clades also vary depending on the combination of aquatic adaptations to be included in the analysis but they tend to be attached to one of the three internodes that connect to the last common ancestor of Sauria.

The present study raises a concern against including marine reptile clades as outgroups in an analysis of the internal phylogeny of a marine reptile clade. One such example would be including *Wumengosaurus* as an outgroup of Sauropterygia, unless future analyses establish that *Wumengosaurus* indeed belongs to Sauropterygia.

## Supporting Information

Text S1 **Data matrix and character descriptions for phylogenetic analysis.**
(DOCX)

Text S2 **Argumentation of aquatic adaptations.**
(DOCX)
