# Supplementary material for: The Enigmatic Marine Reptile Nanchangosaurus from the Lower Triassic of Hubei, China and the Phylogenetic Affinities of Hupehsuchia
Source: PLoS One. 2014 Jul 11;9(7):e102361. doi: 10.1371/journal.pone.0102361 (PMC4094528; doi:10.1371/journal.pone.0102361)
Supplement: Text S1 — Data matrix and character descriptions for phylogenetic analysis. (DOCX) [file pone.0102361.s001.docx]

**Text S1.** Phylogenetic Characters

**Characters adopted from the literature in addition to the 188 characters used by [**[**1**](#_ENREF_1)**]**

The following characters were adopted from [[2](#_ENREF_2)] and added to the 188 characters used by [[1](#_ENREF_1)]. The characters with numbers 118 or smaller in [[2](#_ENREF_2)] were already examined by [[3](#_ENREF_3)], who discarded 37 of the characters. Of these 37, we resurrected character 72 as our character 189. We then screened the rest of the characters and adopted 10 more characters that were not specific to Sauropterygia or Saurosphargidae, or redundant with the characters that already existed in the first 188 characters from [[1](#_ENREF_1)]. In total the following 11 characters were adopted.

189. character 72 of [[2](#_ENREF_2)].

190. character 126 of [[2](#_ENREF_2)].

191. character 131 of [[2](#_ENREF_2)].

192. character 135 of [[2](#_ENREF_2)].

193. character 136 of [[2](#_ENREF_2)].

194. character 140 of [[2](#_ENREF_2)].

195. character 141 of [[2](#_ENREF_2)].

196. character 142 of [[2](#_ENREF_2)].

197. character 145 of [[2](#_ENREF_2)].

198. character 148 of [[2](#_ENREF_2)].

199. character 149 of [[2](#_ENREF_2)].

**New characters**

The following new characters were added to the 199 characters resulting from [[1](#_ENREF_1),[2](#_ENREF_2)].

200. Premaxilla is longer than maxilla: (0) false; (1) true.

201. Nasal extends anteriorly much beyond external naris: (0) false; (1) true.

202. Frontal, butterfly-shaped with antero- and postero-lateral processes: (0) false; (1) true.

203. Supratemporal occipital 'lappet': (0) absent; (1) present.

204. Large scleral ring filling the orbit: (0) absent; (1) present.

205. Angular maximum exposed height relative to surangular: (0) subequal or angular higher; (1) angular clearly lower.

206. Body compression: (0) subequal or slightly compressed dorso-ventrally; (1) clearly flattened dorso-ventrally; (2) bilaterally compressed.

207. Lateral gastralia, expanded and flat: (0) false; (1) true.

208. Strong anticlination of mid-caudal neural spines: (0) absent; (1) present.

209. Humerus anterior flange: (0) absent; (1) present.

210. Radius anterior flange: (0) absent; (1) present.

211. Radiale larger than other carpals: (0) false; (1) true.

212. Forelimb digits: (0) at least some divergent; (1) all convergent.

213. Femur, distal and proximal extremities: (0) of subequal widths; (1) distal one much wider.

**Modified characters**

The following characters were modified from [[1](#_ENREF_1)].

55. Interclavicle anterior process or triangle: (0) present; (1) absent. See the main text for explanation.

64. Ectepicondylar groove: (0) open and notched anteriorly; (1) open without anterior notch; (2) closed (i.e., ectepicondylar foramen present); (3) absent. State 3 was added. This was necessary because the groove is completely absent in ichthyopterygians and hupehsuchians, and probably in *Wumengosaurus*.

152. Premaxilla dentition: (0) 4 or more; (1) 3 or less; (2) edentulous. Sate 2 was added.

154. Frontal anterior margins: (0) frontal suture with nasal transverse; (1) oblique, pointing antero-laterally; (2) oblique, pointing antero-medially. Sate 1 was modified and State 2 was added. These two states cannot be considered homologous although both are 'oblique'.

186. Dorsal vertebral count: (0) 19 or more; (1) 18 or less. The threshold was determined based on histograms derived from the data in [[4](#_ENREF_4)], where basal amniotes have at least 19 dorsal vertebrae. This value also matches the difference between 24 and 5, the former being the threshold used for the original coding for presacral count, the latter for the cervical count (character 187). See the main text for further explanation.

191. Median gastral element: (0) angulated, pointing anteriorly; (1) straight; (2) angulated, pointing posteriorly. State 0 was emended, and state 2 was added.

**Emended character states outside of Hupehsuchia**

The following character states were emended relative to [[1](#_ENREF_1)] or [[2](#_ENREF_2)].

8. Preorbital and postorbital region of skull: modified to "0" in *Sinosaurosphargis* and *Largocephalosaurus*.

26. Quadrate: modified to "0" in *Wumengosaurus*.

35. Retroarticular process: modified to "0" in *Largocephalosaurus*.

64. Ectepicondylar groove: modified to "3" in *Wumengosaurus*, ichthyopterygians, and hupehsuchians.

65. Entepicondylar foramen: modified to "1" in *Simosaurus*.

67. Iliac blade: modified to "1" in Pistosaurids.

129. Lacrimal duct: modified to "?" in sauropterygians, which lack the lacrimal. The absence of lacrimal is already reflected in character 6, so we refrained from adding a state.

155. Frontal anterior margin: modified to "2" in ichthyopterygians.

181. Lacrimal: modified to "?" in sauropterygians and saurosphargids because the lacrimal is absent. The absence is already reflected in character 6, so we refrained from adding a state.

**Hupehsuchia coding emendations**

2(? to 0); 7(2 to 1); 10(0 to 1); 11(? to 0); 17(0 to 2); 20(0 to 1); 21(1 to 0); 24(0 to ?); 25(? to 0); 30(0 to ?); 31(1 to 0); 32(0 to ?); 33(1 to 0); 34(0 to 1); 39(1 to ?); 40(? to 1); 41(1 to 0); 47(1 to 0); 49(? to 0); 53(? to 0); 54(? to 0); 57(? to 1); 58(? to 1); 64(? to 3); 67(? to 1); 74(? to 1); 86(? to 1); 87(? to 1); 89(1 to 0); 90(0 to 1); 92(1 to ?); 95(2 to ?); 97(1 to ?); 102(1 to 0); 104(1 to 2); 105(? to 0); 106(3 to 2); 110(? to 0); 125(0 to ?); 126(? to 1); 127(? to 1); 128(? to 0); 135(0 to ?); 138(1 to ?); 139(0 to ?); 140(1 to ?); 141(0 to ?); 142(? to 1); 144(? to 1); 150(0 to 1); 151(1 to 2); 154(0 to 1); 156(1 to ?); 158(0 to ?); 162(0 to ?); 174(0 to ?); 175(? to 0); 179)? to 0); 181(? to 0); 183(1 to ?); 186(? to 0); 188(0 to 1).

Total: 62 out of the original 188 characters.

**Raw Character Matrix**

Seymouriidae

000000100000000000000000000000000000000000000000000000000000000000000000000000000?0000000??00000100100000010000000000100101000000000000000000000000000000000000000(01)00000000000000?0000001000?0?000(01)010000000?0?000000

Synapsida

000(01)000(01)000100001000(01)0000000000000(01)0000000000000000000000000000000000000000000000?0000001?00000010(01)000100(02)100000(01)0000(01)(01)00010000(01)00110000000000(01)000000000000000000010000000000000(01)?000000(01)(01)11?0?000?00000000000?000000

Parareptilia

00000(01)(01)(01)0(01)0(01)0000(01)(01)00020000000010001(01)(01)0(01)000000000(01)00000(01)0?000000(012)0000(01)000100000000?0000001??00000100000000(01)100000(01)000000000000(01)10010100(01)(01)(01)0(01)(01)(01)0(01)000(01)000(12)000(01)00000(01)(01){01}(01)(01)(01)0(01)1010000(12)0?(01)00010(01)(01)(01)(01)?0?0(012)0?0(02)00000000(01)?000000

Captorhinidae

00000000000(01)0010000000?000000010010000000000?00000?000000000000000000000000000000?1000001??1???01000?0000210101000000000000000100001000000000000000000000001000000000000000000020?0000111000000000000000000000?000000

Araeoscelidia

0000000131000000(01)0000010000?0010000010000000?00000000010000000000000000000000000100000001000010000000010011111100000000000000010?00100000?010000000000100001000??00000000000110(12)000000111011000000000000000000?000000

Rhynchocephalia

0(02)000(12)001(01)(01)(01)(02)11(01)(12)001(01)010(01)111(01)0100011131(01)0(01)01000001111010(01)000000200011110110(01)101110000000111(01)0(01)20110(01)0001(01)(01)1(12)0000(01)00000000000010111(01)11101(01)11011110(01)0010(01)000011(01)01002000111111(01)0010010?(01)110111000000010000000010?000000

Squamata

00000(12)(01)(012)10(01)(012)(02)110221?(01)(012)(01)(01)(01)1111010(01)0111311(02)10(01)00(01)0011(01)101(01)1000000(02)100111101101101110000000(01)(01)(12)1(01)01(01)11(01)(01)0001011(12)1100(01)0(01)0000000000(01)0(01)1100110111(01)110011(01)(01)1(01)120(012)0011001(01)021(01)(01)1(01)1111100100100?11(01)(01)1(01)0000(02)0010(01)00000010?000000

Prolacerta

010101013100011020010010011?0010001010110010?00101110000100000001200111010101011000011000010?01011001111121200101000000000000010?00011011100011?01111010010111010010000110201212100001111?1100?000?00000000010?000000

Trilophosaurus

01000?11100231100?0??2?0?110001000111011(12)010?0010111001010000?000000111010101011?100011001100??011110111011000?111???0000000?????????1011101110101001012010?1111102110?1?020101211?0??1???110000?0000?000000??????000

Rhynchosauria

01000100101231112101100001110010101112110010?001011100101?0000001000111011101011001111110111101011001111130201111001000000001100?1101100110011110110101200011110?0200111112011111110011111110000?0000000000010?000000

Archosauriformes

0101010(01)1(01)(01)(02)(02)(01)101(12)00(01)(012)10(01)11?0(01)10(01)(01)1(01)1111(012)(01)(01)001(01)1(02)(01)1100101(01)0000(01)0100011(12)(01)1(01)1(01)101110(01)0111100111(01)1111001112120(12)00101(01)0(01)0010000001(01)(01)(01)010111?1110111?0(01)111010000?1(01)1110(12)0100?11200011?(01)000111011(012)0000(12)(01)000000000010?000000

Claudiosaurus

0000010111000110200110?000??00100000101?0000?00101110010100011110000111110000000?00010?01120012011000101011000000000000000000?11??11??10??010111011100100?01100??010?000000001020000101101100000000000000000100000000

Coelurosauravus

10010??0100?011021010????11?0?????10?31?0110000001?????????000000000?1?100000000000000?0112??????????10001?11???00??00?0?0???111??0?11???1??????0000101?000111??????????000000021010?0110112?0?00?0????00000?0???????

Kuehneosauridae

010001001102011022011010111110100010?0110110?11?0111101000000012100112011?1???10000110?010200??000001002011?000000300010?00001010000??01??10??0??00?10?00001100?0010001?1000?002000010110?1?0000000?01000000100?????0

Acerosodontosaurus

??????013??????0201??????0??0????0?1??1000?0?00?0?0????????0??00020??????????????0?????0?10???????????01?0??0????????0???0????100??0?????????????111??????0????????????1?????0???0??0?1?0??0????????????????????00???

Tangasaurus

?????????????????????????????????????????????0??0??????????000000??????????01???1??????????????????????1?0?2??????????????0?????????????????0???01?0?????????????????????????????????????10????????????????????????0?

Youngina

00000101310000001000001000??0??0001???1000?000??0?????10???0??000?10?1??????0000100000?01110010?1?00??0110????100?00000?0000?000001?1?????10????01?????00?01??????1???0?1???1?0??0?000?10(01)00???000010000000000?000?0?

Thadeosaurus

???????????????????????????????0????????00?0?0?001????10?00000020200?1011???00?01???????????????1??0??0110??0??00?????00??0?????????????????????0110101???????????1??????00?100??????????0?000?0??000????????00000000

Lanthanolania

000?01013??????02??????????????0?0??????????????????????????????????????????????????????1?1?????0?????????????????????????????00????????????????????????0?0???????2???????????????0?0?1?1???????????????0000?????????

Orovenator

00000001310{02}1???{12}???0????0??0010?0?010100?????????????????????????????????????????000100101?00000?00???????????????000???0?000?100??000???00000????????000010????000000?????????10000??????????????????000001????????

Sophineta

0000011010000110200100???11?1?1000?013100??1000??0?????????????????0?????????????00000?00120000????0??01??1????????000???0?001011110???????0???????????000011??????1????0??0010??21000?1?????????????????????????????

Pamelina

010001001102011022?110???1111?10001010110??00111?1???????????????????????????????001100010200??1??00??02?10???????300????0?00101?000???????0???????????000111?????1?0???1????0??000010?1?????????????????????????????

Tanystropheus

1101010130210110221?0010011?0010001010110110?00101110000100000001101111010011011000011?0111000211111010200100010100000000000011??00011111100011?0111101001011101?02000011020021210100?11111200000(012)010000000010?000000

Choristodera

110101021002111010(01)01110111?0?101010101111001001101100101000000(02)1210111110101011?1011(01)000(01)1100001100000101(01)10001(01)10000000000?0101?1?1100111010?101111?1010011101?01100?1100010011010001?01100000000?00000000?1?000000

Macrocnemus

110101013102111021???0??01011010001010110110?001011100001000??0?12011110100010110?0?11?0001????11?0101?11001001010?000000000?11???001101?1?00?1?011010100?0111????1??0011020121210100?11011?00000?010?000000100000000

Hovasaurus

??????0?3?0?0??0201?0210?011???????????000?0?00?00010?1??0000002020??101?000?00?10???????10???????00?1?102?200??0?0???00??0???????01010?????????0111101??10??????????????00?100?00????1??000?0000?000????1???0??0??00

Pachypleurosaurs

1000(01)20131000110200110?1011101010110?11101111001101001(01)2111101(01)(01)0(12)11111111011100000010?01120????1?0100020(01)12001?01?0002100000100??11?????11?11??11110020000111011121011?00000001(01)0?1?1111(01)12000100010000010011?000000

Simosaurus

10001202202111102001111000100101001011111111101110100112111101111211112111011100000010?01120002?111111020?1?000001?0112110000101??11?????10?11??11110020000111011121011?0000000100?1?1110?120001000100000000?1??000??

Placodus

1011021021(02)101102000101001110100101101100110011100110111111111101211111111011100000010?01101?010110101020211000000?1100001011110?101???1?121101?111110?110111001112110010000000110?1??111012001211010000000001?000000

Pistosauridae

1000120121113110201?11?010110100?0???1011??110?1??1101??11111111121??1??????????000011?01110?0??1111?102?01?00??1??0112100000101??11?????11?11??11110020000111011121011?0000000101?1??110?10(01)10(012)(01)0(01)(01)00010000?1?000000

Askeptosaurus

1010110130000110211?011?111?10100001101101101001001000001000011011001111100010110000010000101120111101010210000001100000000001100?101101?1?10?00011110200101110??020011?0000000211010?11001001??010??0010100100000000

Clarazia

10001?0001000110221?00??111?10101011131?0110000?00110?001?0?0????20??1111?0111100?1001?01?11?1?011110101001100000111000011010110??1??????111??0???101??00101?1????00?00?0000000?1??1??1000??00????0??0000100??0?000?1

Thalattosaurus

10001?0131000110221?01??111?1?10101112110?001000?0???????00001???????0111????????01011?01?11?1???1100?01?????0???111001011010110?110?????1?1??0???1????00101?1????200011?????0??1??1??100?1????????????10?001????????

Helveticosaurus

100???003????????????0?????????????0?00?0110?101?01100101000110?111010211??111100?1000?011??????????0102?301000001?00001?000?1?1?????????????????1111?20?????????????00?10?0000100100?11????001201000?00????00?000000

Largocephalosaurus

1000020031(02)311100201?1??00010012?00000110111?100(01)011011211?1211(02)1111?001110(01)100000001110012?????????0??2?1000000(01)1100?21?000?1?0??11???????1?10011111??(01)01010????02??00?1?0???021011???10010110010(23)020100000?10000000

Sinosaurosphargis

10000?00412301100201?1??0?0?00?2?000101?01???2??00110112?1?1211??1????1??????????0001110??2?0020??110??2?10?0??????001??00?0?100??01????1??1?1????10???001110????020?0??????????1011????0?001100220?20100000?11?0000?

Wumengosaurus

1010010131???110210110???01?0?????10??110110?0001010110011?001130211?121110??10000000110012?????????0?020310000011?00021?000?110??1???????????0?11110??000???1???????00?0?000?020001011?001000010?00?0011(01)?1000000000

Nanchangosaurus

100?010131010110210100?0000?0?????10???1??100000001100??11001113121??0211????0??0000011?012?????????0002021100??010???0??00??1?0??00?????????1?1111????2011?1????????0??0??????2000101?1101?11201?0?1???0110?2101?100

Hupehsuchus

1001011131010110210100??000?0?0??110???101100000001100??110011131211?021110010000000011?012???????110002021100??010???010000?110??00?????????1?111110122011?0?????21?0??00000?02000101?1101111201?0110??0110021011100

Chaohusaurus

1011011131000110210100??001?0?00?100?01101100200(01)0100002110011131211?02111011(01)1000000110012????01?1101010010000?010000010100?110??00???????1?1?1111100(012)002011????02??00?00000?0201010110100(01)01000?0000011011120111011

Utatsusaurus

1011010111010110010101??000???00?10010110110?2?010110000110011131201?021110010100?001110012?????1?11000100100000010000010000?110??00???????1?10?1111012002111?????10?01?00000?0211010111101(01)01000?000001101112011?011

**Marinereptilepartofcharactermatrixwithaquaticadaptationscodedas"?"**

Pachypleurosaurs

?000(01)201?1000110200110?1011101010110?111011110011?1001(01)21?110??(01)0(12)??11111?011100000010?01120????1?0100020(01)12001?0??0002?00000100??11?????11?11???11100?0000111011121011?00?00001(01)0???1111(01)12000100010000010011?000000

Simosaurus

?000120220211110200111100010010100101111111110111?1001121?110??112??11?11?011100000010?01120002?111111020?1?00000??0112?10000101??11?????10?11???11100?0000111011121011?00?0000100???1110?120001000100000000?1??000??

Placodus

?011021021(02)10110200010100111010010110110011001110?1101111?11???012??11111?011100000010?01101?010110101020211000000?1100001011110?101???1?121101??11110?1101110011121100100?0000110????111?12001211010000000001?000000

Pistosauridae

?000120121113110201?11?010110100?0???1011??110?1??1101??1?11???112???1??????????000011?01110?0??1111?102?01?00??1??0112?00000101??11?????11?11???11100?0000111011121011?00?0000101????110?10(01)10(012)(01)0(01)(01)00010000?1?000000

Askeptosaurus

?0101101?0000110211?011?111?101000011011011010010?10000010000??01100111110001011000001000010112011110101021000000?100000000001100?101101?1?10?00011110?00101110??020011?00?00002110?0?110?1001??010??0010100100000000

Clarazia

?0001?0001000110221?00??111?10101011131?0110000?0?110?001?0?0????20??1111?0111100?1001?01?11?1?011110101001100000?11000011010110??1??????111??0???101??00101?1????00?00?00?0000?1?????100???00????0??0000100??0?000?1

Thalattosaurus

?0001?01?1000110221?01??111?1?10101112110?001000?????????0000????????0111????????01011?01?11?1???1100?01?????0????11001011010110?110?????1?1??0???1????00101?1????200011?????0??1?????100?1????????????10?001????????

Helveticosaurus

?00???00?????????????0?????????????0?00?0110?101??1100101000??0?11?010?11??111100?1000?011??????????0102?30100000??0000??000?1?1?????????????????1111??0?????????????00?10?0000100100?11????001201000?00????00?000000

Largocephalosaurus

?0000200?1(02)311100201?1??00010012?00000110111?100(01)?1101121??1???(02)11???0011?0(01)100000001110012?????????0??2?1000000(01)?100?2??000?1?0??11???????1?100?1111??(01)01010????02??00?1?????02101????10?10110010(23)020100000?10000000

Sinosaurosphargis

?0000?00?12301100201?1??0?0?00?2?000101?01???2??0?110112???1?????1????1??????????0001110??2?0020??110??2?10?0??????001??00?0?100??01????1??1?1????10???001110????020?0??????????101?????0?001100220?20100000?11?0000?

Wumengosaurus

?0100101?1???110210110???01?0?????10??110110?0001?1011001??00??302???1?11?0??10000000110012?????????0?02031000001??0002??000?110??1???????????0??1110??000???1???????00?0??00?02000?011?0?1000010?00?0011(01)?1000000000

Nanchangosaurus

?00?0101?1010110210100?0000?0?????10???1??1000000?1100??1?00???312???0?11????0??0000011?012?????????0002021100??0?0???0??00??1?0??00?????????1?1?11????2011?1????????0??0??????2000?01?11?1?11201?0?1???0110?2101?100

Hupehsuchus

?0010111?1010110210100??000?0?0??110???1011000000?1100??1?00???312???0?11?0010000000011?012???????110002021100??0?0???0?0000?110??00?????????1?1?11101?2011?0?????21?0??00?00?02000?01?11?1111201?0110??0110021011100

Chaohusaurus

?0110111?1000110210100??001?0?00?100?01101100200(01)?1000021?00???312???0?11?011(01)1000000110012????01?1101010010000?0?00000?0100?110??00???????1?1?1?11100?002011????02??00?00?00?02010?01101?0(01)01000?0000011011120111011

Utatsusaurus

?011010111010110010101??000???00?10010110110?2?01?1100001?00???3120??0?11?0010100?001110012?????1?110001001000000?00000?0000?110??00???????1?10??11101?002111?????10?01?00?00?02110?01111?1(01)01000?000001101112011?011

**References**

1. Reisz RR, Modesto SP, Scott DM (2011) A new Early Permian reptile and its significance in early diapsid evolution. Proceedings of the Royal Society B-Biological Sciences 278: 3731-3737.

2. Li C, Jiang D, Cheng L, Wu X, Rieppel O (2013) A new species of *Largocephalosaurus* (Diapsida: Saurosphargidae), with implications for the morphological diversity and phylogeny of the group. Geological Magazine FirstView: 1-21.

3. Müller J (2004) The relationships among diapsid reptiles and the influence of taxon selection. In: Arratia G, Wilson MVH, Cloutier R, editors. Recent advances in the origin and early radiation of vertebrates. Munich, Germany: Verlag Dr. Friedrich Pfeil. pp. 379-408.

4. Mueller J, Scheyer TM, Head JJ, Barrett PM, Werneburg I, et al. (2010) Homeotic effects, somitogenesis and the evolution of vertebral numbers in recent and fossil amniotes. Proceedings of the National Academy of Sciences of the United States of America 107: 2118-2123.
