# Supplementary material for: The Enigmatic Marine Reptile Nanchangosaurus from the Lower Triassic of Hubei, China and the Phylogenetic Affinities of Hupehsuchia
Source: PLoS One. 2014 Jul 11;9(7):e102361. doi: 10.1371/journal.pone.0102361 (PMC4094528; doi:10.1371/journal.pone.0102361)
Supplement: Text S2 — Argumentation of aquatic adaptations. (DOCX) [file pone.0102361.s002.docx]

**Appendix: Aquatic Adaptations**

**Eighteen aquatic adaptations that bias the present analyses**

**Premaxilla large (character 1, state 1)**. This feature results in an elongated snout, which has long been considered an aquatic adaptation [[1](#_ENREF_1)]. It is a character state found only in aquatic reptiles and *Coelurosauravus* in our data set. This feature dislocated the external naris posteriorly from the terminal snout position, where the hydrodynamic pressure during swimming is high. See also character 180.

**Upper temporal fossae present and distinctly smaller than the orbit (character 9, state 3)**. This feature is expected in marine reptiles for a combination of two reasons. First, the need to support the mandible diminishes in water, reducing the mass of jaw adductor muscles. Second, the eyes are expected to become large in the marine environment to collect more light. The first reason may be reversed if the lifestyle of a marine reptile requires a stronger biting force than usual, as in *Placodus*, or the jaw is extremely long, as in many ichthyopterygians. It is found in most marine reptiles except durophagous forms, some large sauropterygians, and *Utatsusaurus*. The feature appears as a synapomorphy of all marine reptiles when analyzing the raw data. However, it is doubtful that the slit-like upper temporal fossa of Thalattosauria is homologous with the small and round fossa of pachypleurosaurs. Character definition probably needs to be revised.

**Sacral (and caudal) ribs of transverse processes to their respective centrum sutured (character 50, state 0).** This is a feature that is expected in aquatic animals that do not need to support the body with the hind limb. It is found in all 15 marine reptiles when known. The same character state is found in basal amniotes, which presumably had weaker body mass support through the hind limb than in more derived forms. The feature appears as a synapomorphy of all marine reptiles when analyzing the raw data.

**Coracoid foramen lies between coracoid and scapula (character 58, state 1)**. This results from the reduction of ossification in the pectoral girdle, which does not have to support the body mass in aquatic vertebrates. It is known in all marine reptiles in the data except thalattosaurs.

**Deltopectoral crest reduced (character 61, state 1)**. This results from the lack of necessity to support the body mass with the forelimb in water. Those marine reptiles that use the forelimb for propulsion may retain the feature. The feature is found in all aquatic reptiles except some sauropterygians, thalattosaurs, and *Wumengosaurus*.

**Insertional crest for latissimus dorsi muscle reduced (character 62, state 1)**. This is another feature that results from the lack of necessity to support the body mass with the forelimb in water. It is found only in all 15 marine reptiles and *Claudiosaurus*, which may have been amphibious [[2](#_ENREF_2)]. The feature appears as a synapomorphy of all marine reptiles when analyzing the raw data.

**Humerus epicondyle reduced (character 63, state 1)**. This is yet another feature that results from the lack of necessity to support the body mass with the forelimb in water. It relates to the size and strength of the elbow articulation.

**Iliac blade reduced (character 67, state 1)**. The reduction of iliac blade indicates a weaker connection between the vertebral column and hip, as well as less hip muscles. Both of these are expected from reduced need for body support using the hind limb. It is found in all marine reptiles except thalattosaurs and *Utatsusaurus*.

**Thyroid fenestra present (character 68, state 1)**. The thyroid fenestra emerges from the reduction of ossification of the pelvic girdle. One of the reasons for such a reduction is the reduced need to support the body mass with the hind limb in water. It is found in most marine reptiles except thalattosaurs and *Helveticosaurus*.

**Intertrochanteric fossa rudimentary or absent (character 71, state 3)**. This feature is expected from the reduced elbow size, as with character 63.

**Foramen for the supposed passage of the pes artery absent between astragalus and calcaneum (character 74, state 1)**. The absence of the passage indeed cannot be confirmed in many marine reptiles because their astragalus and calcaneum are only partly ossified, leaving much space between them. Such a reduction of ossification is expected from the reduced need to support the body mass with the hind limb in water. It is known in all marine reptiles except thalattosaurs.

**Processus lateralis (of the pubis) is absent (character 114, state 1)**. The process disappears with the reduction of ossification of the pelvic girdle, which is expected from the reduced body support using the hind limb in water. It is found in all marine reptiles except *Placodus*. The feature appears as a synapomorphy of all marine reptiles when analyzing the raw data.

**Internal trochanter is reduced (character 120, state 1)**. This results from a reduction of puboischiofemoralis muscle, and is expected to be associated with the reduction of the pelvic girdle. It is known only in aquatic reptiles in the data set, and found in all marine reptile except thalattosaurs and *Placodus*.

**Humeral torsion: angle between opposing ends reduced to not more than 20° (character 145, state 1)**. The angle reduction may occur with the change of limb posture for many reasons. One such change is the formation of flippers or paddles that have reduced needs to support the body mass. It is found across all marine reptiles except thalattosaurs.

**Number of pedal centralia: both centralia lost (character 151, state 2)**. The centralia are lost through skeletal paedomorphosis, which is common among marine reptiles. The reduced need for body mass support by the ankle is expected to lead to the reduction of tarsal numbers. It is found in all marine reptiles in the data except *Chaohusaurus*. The thalattosaur *Endennasaurus* is also known to possess at least one pedal centrale, although it is not in the data set [[3](#_ENREF_3)]. See [[4](#_ENREF_4)] for the discussion of a problem with this character. The feature appears as a synapomorphy of all marine reptiles when analyzing the raw data.

**Astragalus and calcaneum never fused in adults (character 171, state 0)**. As with character 74, this character is expected in marine reptiles—reduced ankle ossifications arising from lessened need for body support in water would result in this feature. It is found in all marine reptiles in the data.

**Nares situated in the central or posterior area of the antorbital skull portion (character 180, state 1)**. Despite the great difference in wording, this character is almost identical to character 1 (premaxilla enlargement)–the nares are displaced posteriorly because of the enlargement of the premaxilla. The two characters are coded differently only for protorosaurs and *Helveticosaurus*, which have a modest premaxillary enlargement. Having both characters would lead to adding weights to the snout elongation in marine reptiles.

**Dorsal count exceeding 18 (character 186, state 0)**—revised from the presacral count as explained in the main text. A presacral count of 36 or more, as originally defined, is found only in those reptiles whose limbs have reduced role in body mass support, such as marine reptiles. A dorsal count of 19 or more is a plesiomorphy of amniotes that reappeared in marine reptiles, as the need for body support with limbs diminished. It is found in all marine reptile except a limited number of pachypleurosaurs.

**Six additional aquatic adaptations that are not as common**

**Parietal(s) in adults paired (character 11, state 0)**. This is an example of skeletal paedomorphosis in marine reptiles discussed by [[5](#_ENREF_5)]. It is a plesiomorphy of amniotes that are modified in those species with strong bite forces. It does not affect the analysis because it vastly prevails in terrestrial species too.

**Distal femoral condyles do not project markedly beyond shaft (character 72, state 1)**. This state is expected in marine reptiles with flippers and paddles because of the reduction of force on the knee, as well as knee motion range—enlarged condyles would stabilize the knee during bending motions and transfer force stably for body support. It is seen in all marine reptiles in the data. It does not seem to affect the outcome of the analysis for an unidentified reason.

**Total number of tarsal ossifications less than four (character 78, state 1)**. As with characters 74 and 171, this character state is expected in marine reptiles because reduced ankle ossifications arising from lessened need for body support in water would result in this feature. It is found in all marine reptiles except *Askeptosaurus*, ichthyopterygians, and hupehsuchians.

**Proximal caudal neural spines tall (character 106, states 2 and 3)**. Tail-based propulsion during swimming would require a large cross-section of muscles in the tail stock. However, these character states are not necessarily found in tail propulsors.

**Total number of carpal ossifications two or three (character 192, states 1 and 2)**. As with characters 74 and 171, these character states are expected in marine reptiles because reduced carpal ossifications arising from lessened need for body support in water would result in these features. However, only about half of marine reptiles have these features.

**Premaxilla longer than maxilla (character 200, state 1**). Although snout elongation is expected in marine reptiles, as with characters 1 and 182, extensive elongation as in this character state is not prevalent across these reptiles.

**References**

1. Carroll RL, Dong Z (1991) *Hupehsuchus*, an enigmatic aquatic reptile from the Triassic of China, and the problem of establishing relationships. Philosophical Transactions of the Royal Society of London Series B-Biological Sciences 331: 131-153.

2. Carroll RL (1981) Plesiosaur Ancestors from the Upper Permian of Madagascar. Philosophical Transactions of the Royal Society of London Series B-Biological Sciences 293: 315-&.

3. Müller J, Renesto S, Evans SE (2005) The marine diapsid reptile *Endennasaurus* from the Upper Triassic of Italy. Palaeontology (Oxford) 48: 15-30.

4. Motani R, Jiang D, Tintori A, Rieppel O, Chen G, et al. (in review) First evidence of centralia in Ichthyopterygia reiterating bias from paedomorphic characters on marine reptile phylogenetic reconstruction. Journal of Vertebrate Paleontology.

5. Rieppel O (1989) *Helveticosaurus zollingeri* Peyer Reptilia Diapsida Skeletal Pedomorphosis Functional Anatomy and Systemic Affinities. Palaeontographica Abteilung A Palaeozoologie-Stratigraphie 123-152.
